# Role of Deubiquitinases in Parkinson’s Disease—Therapeutic Perspectives

**DOI:** 10.3390/cells12040651

**Published:** 2023-02-17

**Authors:** Pernille Y. Ø. Nielsen, Justyna Okarmus, Morten Meyer

**Affiliations:** 1 Department of Neurobiology Research, Institute of Molecular Medicine, University of Southern Denmark, 5000 Odense, Denmark; 2 Department of Neurology, Odense University Hospital, 5000 Odense, Denmark; 3 BRIDGE—Brain Research Inter-Disciplinary Guided Excellence, Department of Clinical Research, University of Southern Denmark, 5000 Odense, Denmark

**Keywords:** neurodegeneration, PARK2, parkin, mitochondria, protein degradation, ubiquitin proteasome system, mitophagy, α-synuclein, Lewy bodies

## Abstract

Parkinson’s disease (PD) is a neurodegenerative disorder that has been associated with mitochondrial dysfunction, oxidative stress, and defects in mitophagy as well as α-synuclein-positive inclusions, termed Lewy bodies (LBs), which are a common pathological hallmark in PD. Mitophagy is a process that maintains cellular health by eliminating dysfunctional mitochondria, and it is triggered by ubiquitination of mitochondrial-associated proteins—e.g., through the PINK1/Parkin pathway—which results in engulfment by the autophagosome and degradation in lysosomes. Deubiquitinating enzymes (DUBs) can regulate this process at several levels by deubiquitinating mitochondrial substrates and other targets in the mitophagic pathway, such as Parkin. Moreover, DUBs can affect α-synuclein aggregation through regulation of degradative pathways, deubiquitination of α-synuclein itself, and/or via co-localization with α-synuclein in inclusions. DUBs with a known association to PD are described in this paper, along with their function. Of interest, DUBs could be useful as novel therapeutic targets against PD through regulation of PD-associated defects.

## 1. Introduction

### 1.1. Parkinson’s Disease

Parkinson’s disease (PD) is a common neurodegenerative disorder primarily affecting individuals over the age of 65 years [1]. Hallmarks of the disease include bradykinesia, resting tremor, and rigidity, which are believed to be caused by the progressive loss of dopaminergic neurons in the substantia nigra pars compacta (SNpc) located in the midbrain. Additionally, PD is associated with non-motor dysfunctions such as sleep disorders, dementia, anxiety, pain, fatigue, and cognitive impairments [2,3]. Although age is the greatest risk factor of the disease, an interplay between environmental and genetic factors is also believed to be involved. Genes associated with rare monogenetic forms of PD account for 5–10% of cases and include PARK2 (Parkin), PTEN-induced putative kinase 1 (PINK1), DJ-1, SNCA (α-synuclein), LRRK2, VPS35, and GBA amongst others. The remaining cases are sporadic with no clear or single cause [4].

Early diagnosis of PD is complicated as it is estimated that motor dysfunctions appear when approximately 70% of the dopaminergic neurons are lost. The resulting reduction in dopamine levels is believed to be the primary cause of symptoms [5]. This cellular loss has been associated with prior events such as oxidative stress and mitochondrial dysfunction, which might be key elements in disease progression and pathogenesis [6]. Mitophagy, the selective degradation of mitochondria, is an important process that prevents and limits the oxidative stress that follows mitochondrial dysfunction. The process is initiated by the accumulation of PINK1 to the outer mitochondrial membrane due to damage or depolarization, which triggers Parkin translocation, Parkin-mediated ubiquitination of mitochondrial membrane proteins, and degradation of the organelle in lysosomes [7]. This process is described in greater detail in later sections. A common neuropathological finding in the post-mortem brains of PD patients is α-synuclein-containing inclusions termed Lewy bodies (LBs). Mutations in the SNCA gene, which encodes α-synuclein, can lead to overexpression due to gene dosage changes, post-translational modifications, and higher prevalence of the monomeric form of the protein, which in turn results in the formation of oligomers and fibrils. Aggregation can also be caused by mitochondrial and proteasomal dysfunction. These changes result in prion-like properties that allow spreading of the protein between interconnected neurons and disruption of several cellular functions such as neurotransmitter release, mitochondrial function, and protein transcription [8]. However, the exact physiological role of α-synuclein and its mechanistic association with PD is still unclear [9,10].

PD treatment is based on symptom management with no available options for preventing or slowing the disease progression. Dopamine-based drugs are often used for the motor dysfunctions, and one of the most widely used is the dopamine precursor Levodopa [11]. The absence of a cure for PD prompts further investigation of the pathogenesis of the disease and the identification of novel therapeutic targets.

### 1.2. Protein Degradation Pathways

#### 1.2.1. The Autophagy–Lysosomal Pathway

Two major degradative pathways are present in the eukaryotic cell: (i) the lysosomal proteolysis pathway, which includes macroautophagy, microautophagy, and chaperone-mediated autophagy (CMA), collectively referred to as the autophagy–lysosome pathway (ALP) and (ii) the ubiquitin–proteasome system (UPS) [12,13].

**Figure 1:**
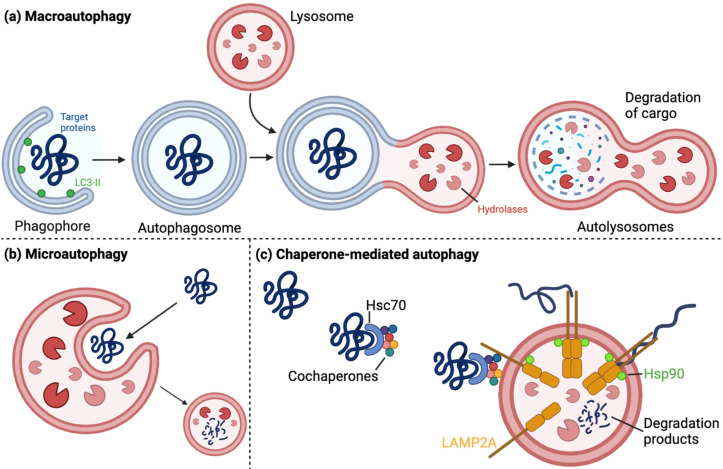
Visual representation of the autophagy–lysosomal pathway. (**a**) Macroautophagy is the engulfment of target proteins by a phagophore through the interaction between LC3-II on the phagophore and the target. The resulting double-membrane autophagosome can then fuse with hydrolase-containing lysosomes, resulting in the lysosomal degradation of the target proteins. (**b**) Microautophagy is the direct endocytosis of target proteins by lysosomes, which results in target degradation. (**c**) Chaperone-mediated autophagy involves several chaperones such as Hsc70, which facilitate the binding of target proteins to lysosomal-associated proteins such as LAMP2A. Subsequent steps include multimerization of LAMP2A proteins, which is stabilized by lysosomal membrane-associated Hsp90. This results in the unfolding of the target protein, internalization into the lysosome, and its subsequent degradation. Created using www.BioRender.com (accessed on 10 December 2022).

Lysosomes are degradative, enzyme-containing organelles important in the cellular waste system, and they are implicated in the degradation of products supplied from several degradation systems [14]. Autophagy performs pro-survival functions and is active during both basal and stress conditions, where it generally targets and degrades long-lived, misfolded, and/or aggregated proteins, dysfunctional organelles, and intracellular pathogens [15,16]. As mentioned previously, three types of autophagy are present in the cell: macroautophagy, microautophagy, and CMA (Figure 1) [13,17].

#### 1.2.2. The Ubiquitin–Proteasome Pathway

Several cellular processes are regulated through the degradation of proteins by the UPS. These include DNA repair, cell cycle progression, apoptosis, transcription, and the cellular stress response [15]. Additionally, UPS degrades misfolded or damaged proteins that could otherwise lead to toxic aggregates [18]. The system is based on two consecutive steps: (i) attachment of ubiquitin to the substrate through covalent bonds and (ii) degradation of the substrate by the 26S proteasome complex through recognition of the ubiquitin chains. In the process, free ubiquitin is released and can be reused [19,20].

**Figure 2:**
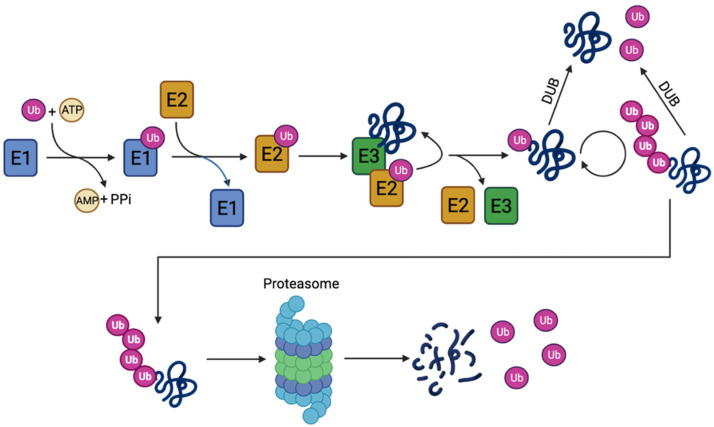
Visualization of the ubiquitin–proteasome system. The E1 ubiquitin-activating enzyme is loaded with ubiquitin through the cleavage of ATP into AMP and PPi. The ubiquitin is then transferred onto the E2 ubiquitin-conjugating enzyme, which can then bind to the E3 ubiquitin ligase. The E3 ubiquitin ligase recognizes the substrate and facilitates the transfer of ubiquitin from the E2 onto the target. This process can be repeated, resulting in the formation of ubiquitin chains. This can be reversed by the action of deubiquitinases (DUBs), or the ubiquitination can lead to the degradation of the target by the proteasome and the release of free ubiquitin monomers. Created using www.BioRender.com (accessed on 10 December 2022).

Ubiquitin is a small protein of 76 amino acids that can be attached to a lysine residue on the substrate protein through an isopeptide bond. The ubiquitination process involves several enzymes: the E1 ubiquitin-activating enzyme, the E2 ubiquitin-conjugating enzyme, and the E3 ubiquitin-protein ligase, as well as the E3 ubiquitin chain-elongating subtype [21]. The process (Figure 2) is initiated by the ATP-dependent, C-terminal adenylation of ubiquitin by E1. This is followed by the formation of a thioester bond between ubiquitin at the C-terminal carbonyl group of glycine and the thiol group of the active site cysteine in the E1 enzyme. Subsequently, the activated ubiquitin can be transferred by a trans-thioesterification to an E2 enzyme, from which the ubiquitin can be attached directly to a lysine residue of a protein substrate through the formation of a complex with E2-ubiquitin and an E3 ligase [22]. The E3 ligases can recognize substrate motifs and hereby confer specificity as well as add additional ubiquitin molecules to the ubiquitin chain [21,23]. Only one ubiquitin-activating E1 enzyme is present in most organisms, including humans, whereas several ubiquitin-conjugating E2s and even more E3 ligases are usually present [24].

Ubiquitin contains seven lysine residues (Lys6, Lys11, Lys27, Lys29, Lys33, Lys48, and Lys63), all of which can be used for chain formation through isopeptide bonds. The ubiquitin chains can be both homotypic, where the same linkage type is used, or heterotypic, including branched chains, where different lysine linkages are present [25]. The different types of linkages can confer different signals, where Lys48 linkages are the most dominant and target the protein for degradation by the proteasome. The other linkages convey other cellular signals [26]. Besides the regulatory potential of the different linkages and their combinations, the removal of the ubiquitin particles by deubiquitinating enzymes (DUBs) adds another layer of regulation to the process [27]. The DUBs found to be associated with PD will be reviewed in this paper.

#### 1.2.3. Mitophagy: A Crosstalk between Autophagy and the Ubiquitin–Proteasome System

For a long time, it was thought that ALP and UPS were independent processes, both critical for cellular homeostasis. However, recent studies have provided evidence of an interplay between the two pathways, with ubiquitin as the common denominator [28]. An example of this interplay is the selective degradation of mitochondria by autophagy, a process termed mitophagy [15]. Mitophagy is important to neuronal health and plays a significant role in neurodegenerative disorders, including PD. The process can be divided into two forms: Parkin-dependent and Parkin-independent mitophagy, where Parkin is an E3 ubiquitin ligase [29,30].

**Figure 3:**
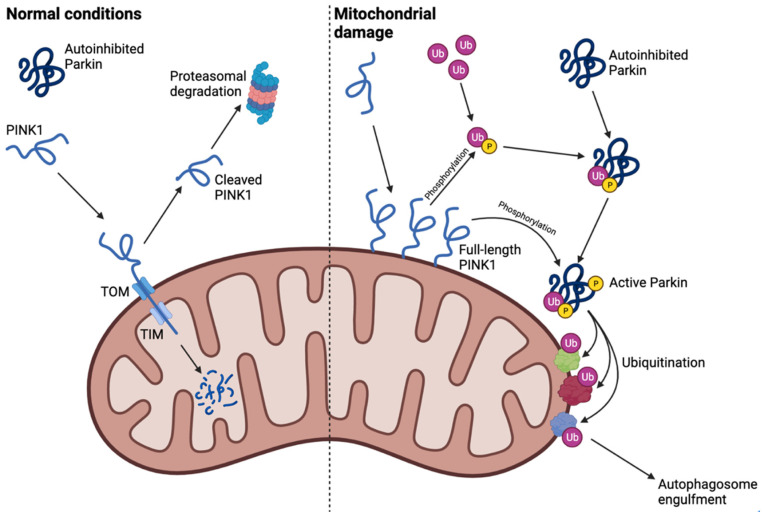
Visualization of mitophagy. Left side: during normal conditions, PINK1 is continuously transported across the mitochondrial membrane through the translocase of inner membrane (TIM) and translocase of outer membrane (TOM), during which cleavage of PINK1 takes place. This results in the formation of a shorter, cleaved form of PINK1 that is targeted for degradation by the proteasome. Low levels of PINK1 are then present at basal conditions, and Parkin is present in its autoinhibited form. Right side: due to mitochondrial damage or mitochondrial depolarization, the cleavage of PINK1 does not occur, leading to its accumulation on the mitochondrial membrane. Accumulated PINK1 can then phosphorylate free ubiquitin, which can bind to Parkin and, along with PINK1-mediated phosphorylation, activate and translocate Parkin to the mitochondria. Here, Parkin can perform ubiquitination reactions on mitochondrial outer membrane proteins, leading to the engulfment of the mitochondria by autophagosomes and subsequent lysosomal degradation. Created using www.BioRender.com (accessed on 10 December 2022).

Under normal conditions, Parkin is mainly found in the cytosol, from where it is recruited to the mitochondria upon mitochondrial depolarization or damage, which results in engulfment by the autophagosome and selective degradation by lysosomes [30,31]. The polypeptide PINK1 is transported across the outer and inner mitochondrial membranes through the translocase of inner membrane (TIM) and the translocase of outer membrane (TOM) complex during basal conditions. This is followed by presenilin-associated rhomboid-like protein (PARL)-mediated cleavage and proteasomal degradation of the protein, which results in low PINK1 expression levels [32]. When the mitochondrial membrane potential is altered (e.g., due to carbonyl cyanide chlorophenylhydrazone (CCCP) treatment, mitochondrial damage, or mitochondrial dysfunction), full-length PINK1 accumulates on the outer mitochondrial membrane [33]. This accumulation is necessary for subsequent recruitment and activation of Parkin. PINK1 then phosphorylates the Ser65 of ubiquitin, which allows phospho-ubiquitin to bind to Parkin. This results in Parkin conformational changes and release of its ubiquitin-like (Ubl) domain that can then be phosphorylated by PINK1 at a homologous Ser65 [31,34,35]. Upon activation and translocation to the mitochondria, Parkin ubiquitinates mitochondrial outer membrane proteins such as Mitofusin 1 and 2 (Mfn1 and Mfn2) as well as voltage-dependent anion channel 1 (VDAC1) [36,37]. The Parkin-dependent ubiquitination results in selective mitophagy and degradation of depolarized mitochondria by engulfment from the autophagosome (Figure 3) [30].

Dysregulation of this pathway or of mitochondrial quality control, e.g., due to mutations in Parkin or PINK1, has been linked to several neurological disorders including PD [38]. As protein ubiquitination is a reversible process, different DUBs can affect this pathway in various ways and affect the progression of PD, thus making these proteins as potential therapeutic targets [39].

## 2. Parkinson’s Disease-Associated Deubiquitinating Enzymes

The human genome is currently known to encode more than a hundred different DUBs with diverse functions [40]. The DUBs can be subdivided into seven families, where six of them are cysteine proteases [41]. Particularly important in relation to PD is the family of ubiquitin-specific protease (USP), but the ubiquitin C-terminal hydrolase (UCH), the Machado-Joseph Disease (MJD), and the ovarian tumor (OTU) domain cysteine protease families also deserve attention.

All members of the USP family contain a catalytic triad consisting of cysteine, histidine, and aspartic acid or asparagine, which cleaves the peptide bond between ubiquitin and the substrate protein [42]. Low sequence homology is observed between the different USP family members apart from this catalytic domain [43], and analysis of the structure of USPs and UCHs suggests that the catalytic triad is only in its active conformation when bound to ubiquitin [42]. Despite the same overall mechanism of action, DUBs differ regarding their target, their function in regulatory protein networks, and their effects on various processes, including mitophagy. Furthermore, phosphorylation of ubiquitin affects the activity of the DUBs in different ways, adding yet another layer of complexity to the regulation [44]. Table 1 lists the DUBs that are related to PD and describes their PD-related function.

### 2.1. USP15 Deubiquitinates Parkin Substrates and Inhibits Mitophagy

USP15, has a wide expression pattern with USP15 mRNA being found in various organs and tissues, including high levels in the pancreas, testes, and thyroid gland [94]. Similarly, it has wide expression across several brain regions including strong expression in midbrain structures and dopaminergic neurons [45].

**Figure 4:**
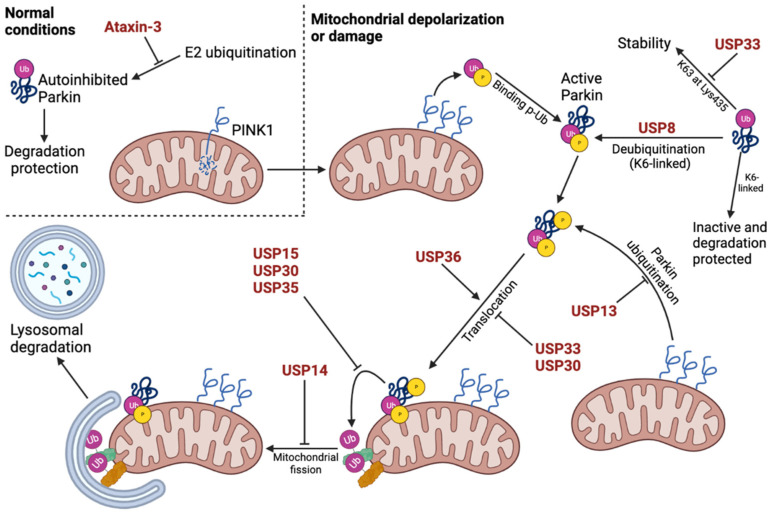
Mitophagy illustration including functions of USP8, USP13, USP14, USP15, USP30, USP33, USP35, USP36, and Ataxin-3. Ataxin-3, USP13, and USP8 inhibit the ubiquitination of Parkin. Ataxin-3 diverts the ubiquitin from the E2 enzyme away from Parkin and onto itself, whereas USP8 removes already formed Lys6-linked ubiquitin chains from Parkin. This ubiquitination of Parkin protects it from degradation, but deubiquitination is necessary for effective translocation and activation of Parkin. Additionally, USP33 also deubiquitinates Lys63-linked chains on Parkin at its Lys435, which decreases the translocation of Parkin to mitochondria and reduces the stability of the protein. USP36 stimulates mitophagy by enhancing the translocation of Parkin. USP15, USP30, and USP35 deubiquitinate outer mitochondrial membrane proteins that have been targeted by active Parkin. These ubiquitinated proteins are essential in the targeted autophagy of the mitochondria during the autophagosome engulfment. The mechanism of action of USP35 is still not clear, but it may stabilize Mfn2 on the outer mitochondrial membrane, where its dissociation from Mfn2 allows Parkin to perform its function. It hereby functions by inhibiting Parkin activity on the mitochondria. Moreover, USP30 and USP33 are believed to inhibit the translocation of Parkin to the mitochondria. However, this role of USP30 is still somewhat controversial. Lastly, USP14 inhibits mitophagy in a PINK1/Parkin-independent manner, but Drp1- and Mfn2-dependent. Here, USP14 inhibits mitophagy by inhibiting the mitochondrial fragmentation prior to autophagosome engulfment. Created using www.BioRender.com (accessed on 15 December 2022).

In a study by Cornelissen et al. [45], USP15 was shown to affect mitophagy and interact with Parkin in Parkin-overexpressing cells. Induced mitophagy was inhibited by co-expression of USP15 and Parkin but could be rescued by USP15 knockdown (KD) or PARK2 KD, suggesting Parkin-dependence. USP15 did not function by deubiquitinating Parkin or affecting its translocation, but rather by deubiquitinating mitochondrial proteins targeted by Parkin with a preference for Lys48- and Lys63-linkages (Figure 4). It has previously been shown that not all PD-related Parkin mutations result in abolished E3 ligase activity [95,96]. Therefore, Cornelissen et al. [45] decided to investigate the role of USP15 in PD patient fibroblasts with Parkin mutations as well as in vivo. Indeed, reduced Parkin levels and non-zero enzyme activity were observed in the PD patient fibroblast, resulting in reduced mitochondrial ubiquitination and clearance that could be rescued by USP15 KD. Comparable results were obtained in PD patient fibroblasts with PINK1 mutations. Similarly, KD of the *Drosophila melanogaster* USP15 homolog in a Parkin KD PD model could rescue several of the defects associated with this model [45,97], hereby showing in vivo implications.

In conclusion, USP15 has been shown to impair Parkin-dependent mitophagy by deubiquitinating mitochondrial proteins targeted by Parkin during mitochondrial stress conditions. This suggest that inhibition of this DUB could be a potential drug target that could be used to rescue impaired mitophagy and/or reduced Parkin activity, but it is yet to be investigated.

### 2.2. USP30 Inhibits PINK1/Parkin-Dependent Mitophagy by Deubiquitinating Mitochondrial Proteins

Deubiquitinase, USP30, has an N-terminal hydrophobic region, which tethers it to the mitochondrial membrane, and the catalytic domain of conserved cysteine and histidine residues facing the cytosol [98]. Similar to USP15, USP30 has been shown to inhibit mitophagy by reversing the ubiquitination of mitochondrial proteins performed by Parkin with preference towards Lys6- and Lys11-linkages (Figure 4). In vitro studies have shown that overexpression of the enzyme results in decreased degradation of mitochondrial proteins TOM20, Hsp60, Mfn1, MIRO, and VDAC as well as reduced recruitment of autophagy markers [46,47,48,49]. In support of this, proteomics analysis identified 41 proteins to be oppositely regulated by USP30 and Parkin. Interestingly, USP30 itself is a Parkin substrate, where USP30 ubiquitination results in its proteasomal degradation [46]. Besides its deubiquitinating role, USP30 may additionally delay mitophagy by regulating the translocation of Parkin to the mitochondria [47], but discrepancies are present between studies regarding this [46,50]. Similarly, some studies have found that ubiquitin phosphorylation reduces USP30 efficiency in removing ubiquitin chains [99], while other studies found that ubiquitin phosphorylation does not influence the efficiency of USP30 nor the function of other DUBs [100]. Lastly, USP30 has been observed to affect mitochondrial function, where its expression influences the mitochondrial membrane potential, the overall oxygen consumption rate, basal respiration, and the oxygen consumption rate during oxidative phosphorylation [46].

In a study by Bingol et al. [46], the function of USP30 was investigated in a *D. melanogaster* PD model with PINK1 or Parkin mutations and simultaneous USP30 KD. Here, USP30 KD largely rescued the mitochondrial morphology defects in the indirect flight muscle of this model and reversed the climbing defects and dopamine deficiency of the PINK1 mutant flies. Similarly, locomotor defects in flies treated with the toxin, paraquat, were also reversed by selective USP30 KD in dopaminergic and aminergic neurons [46]. Further supporting the role of USP30 in PD and its potential as a therapeutic target, several groups have designed, characterized, and investigated the effects and selectivity of a variety of USP30 inhibitors and found some that increased ubiquitination of Parkin targets and mitigated mitophagy [51,52,53,55,56]. Several of the studies showed that these effects are dependent on Parkin expression or that the inhibitors can rescue mitophagy in patient-derived cells with Parkin heterozygosity [53,55]. Similarly, inhibitors of USP30 can increase basal mitophagy in induced pluripotent stem cell (iPSC)-derived dopaminergic neurons with PARK2 knockout (KO) as well as strengthen toxin-induced mitophagy [101]. Here, the inhibitors normalized toxin-induced ROS levels in both control and KO cells as well as reduced basal ROS levels in the PARK2 KO neurons. This was associated with decreased levels of specific pro-inflammatory cytokines at baseline in the mutant cell line [101]. Interestingly, in a study by Yue et al. [54], the small, natural diterpenoid derivative, 15-oxospiramilacetone (S3), was found to inhibit USP30 and induce mitochondrial fusion, rescue mitochondrial membrane potential, increase ATP levels, and rescue mitochondrial DNA (mtDNA) deficiency in cells without Mfn1 or Mfn2. It was found that the expression of OPA1 and at least one of the Mfn proteins was necessary for the function of S3 even though the function was independent of the level of expression of these. However, the function of S3 was dependent on the Cys77 residue in the catalytic domain of USP30, where USP30 KD led to similar effects in cells as the inhibitor [54]. Lastly, Luo et al. [56], showed that their USP30 inhibitor had positive effects on mitophagy in vivo using cardiac tissue without inducing cardiac pathology.

Collectively, USP30 seems to function by reversing Parkin-mediated ubiquitination of mitochondrial proteins, which subsequently leads to impaired mitophagy, accumulation of depolarized mitochondria, and mitochondrial morphology defects associated with in vitro and in vivo models of PD. Several studies on USP30 inhibitors suggest great promise for these as therapeutic drugs in the future.

### 2.3. USP33 Deubiquitinates K63-Linked Ubiquitin on Parkin at Lys435

USP33, also named VHL-interacting deubiquitinating enzyme 1 (VDU1), is closely related to USP20, which is also named VDU2, and they both interact with the von Hippel-Lindau (VHL) tumor suppressor [102]. USP33 is widely expressed in several tissues with high levels in the brain, heart, kidney, and placenta [103]. When investigated in mouse tissue, USP33 is predominantly expressed in the central nervous system (CNS) with high prevalence in the amygdala, frontal and cerebral cortex, nucleus trigeminus, the lower and upper spinal cord, hypothalamus, and SN [104]. USP33 has been shown to function in several pathways including commissural midline crossing of axons [105].

In a study by Niu et al. [57], USP33 interacted with Parkin through the catalytic USP domain of USP33 and the Ubl and RING2 domain of Parkin. This interaction was low at basal conditions but increased upon CCCP treatment [57]. In a previous study, all three isoforms of the protein were mainly localized to endoplasmic reticulum (ER)-associated structures or the Golgi apparatus [106]. However, Niu et al. [57] showed that USP33 co-localized with TOM20 on the outer mitochondrial membrane, and they identified the part of the protein sequence that potentially encodes the transmembrane domain. Here, USP33 was shown to deubiquitinate Parkin with preferences for Lys63-linked ubiquitin chains attached to Lys435 of Parkin. This ubiquitination of Parkin is important in the stabilization of the protein as well as the translocation of Parkin to the mitochondria, where USP33 KD resulted in augmentation of stabilization, translocation, and mitophagy observed as faster TOM20 ubiquitination and removal as well as diminished mtDNA staining. Lastly, Niu et al. [57], showed that SH-SY5Y cells experienced decreased apoptotic cell death when USP33 was depleted compared to control both in the presence and absence of MPTP.

In summary, data suggest that USP33 is a DUB that primarily targets Lys435-linked ubiquitin chains on Parkin, leading to Parkin deubiquitination and a subsequent decrease in stabilization, translocation, and mitophagy. This suggests that inhibition of USP33 could have potential therapeutic relevance in PD, but its wide distribution in the body should also be taken into consideration.

### 2.4. USP35 Inhibits Parkin-Mediated Mitophagy through Mfn2 Stabilization

USP35 is a poorly studied deubiquitinase that is found in two isoforms (UniProt accession number: Q9P2H5). In a study by Wang et al. [47], both short-USP35 (s-USP35) and long-USP35 (l-USP35) were investigated and were shown to interact. Only s-USP35 contains an N-terminal putative mitochondrial-targeting sequence (MTS), which tethers it to the mitochondria in basal conditions. Results suggested that s-USP35 cycles between the mitochondria and the cytosol depending on the mitochondrial membrane potential and that it recruits l-USP35 to the mitochondria when it is located there. This was supported by removal of the MTS, which resulted in cytosol-localized s-USP35 only. However, both WT and MTS deficient s-USP35 had reduced Parkin-dependent mitochondrial clearance, suggesting that USP35 plays a role in Parkin-mediated mitophagy regardless of its subcellular localization. Interestingly, USP35 KD was shown to reduce steady-state levels and the stabilization of Mfn2 on the mitochondrial membrane, although without affecting mitochondrial morphology. However, increased interaction between ubiquitinated Mfn2 and Parkin upon USP35 KD was observed. USP35 was therefore suggested to have a housekeeping role for healthy mitochondria, where its dissociation from damaged mitochondria allows Parkin to associate with Mfn2 and mediate its function in mitophagy (Figure 4) [47].

Overall, USP35 appears to play a dual role in mitochondrial health. Firstly, it deubiquitinates and stabilizes outer mitochondrial membrane protein, Mfn2, which is important for healthy mitochondrial morphology. Secondly, USP35 dissociates from Mfn2 during mitochondrial depolarization, leading to increased interaction with Parkin and increased mitochondrial clearance at a similar level as USP35 KD. To our knowledge, no inhibitors of USP35 have yet been developed, and its potential as a drug target is therefore still unknown.

### 2.5. USP8 Regulates Parkin Degradation and Translocation, α-Synuclein Clearance, and Mfn Levels

The deubiquitinase USP8 has a wide expression in the brain and, most importantly, the SN [107], and it has been shown to have several roles regarding PD. Firstly, in a study by Durcan et al. [50], USP8 deubiquitinated Parkin itself rather than deubiquitinating Parkin targets with preference for Lys6-linked chains. This ubiquitination of Parkin protects it from degradation as USP8-mediated deubiquitination was shown to increase Parkin turnover (Figure 4). However, USP8 deubiquitination was also observed to decrease the autoinhibited state of the protein and was necessary for efficient Parkin recruitment to depolarized mitochondria. USP8 KD delayed but not fully abolished Parkin translocation and mitophagy without affecting mitochondrial membrane potential, steady-state levels of PINK1, PINK1 accumulation on mitochondria, mitochondrial protein levels and ubiquitination, or mitochondrial morphology, fusion, fission, and fragmentation [50].

**Figure 5:**
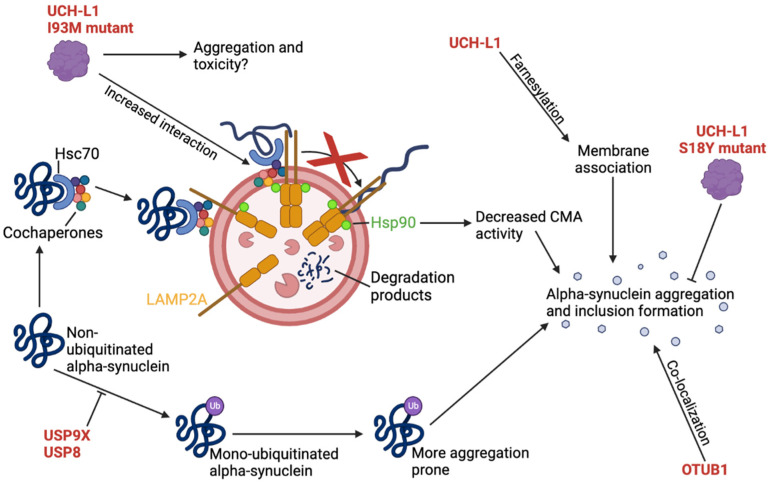
Visualization of how UCH-L1, USP9X, OTUB1, and USP8 affect the CMA and α-synuclein aggregation. The I93M UCH-L1 mutant is a more unfolded variant of the UCH-L1 protein, leading to possible increased aggregation and toxicity. More clearly, however, the mutation results in increased interaction with LAMP2A, Hsc70, and Hsp90, which inhibits the CMA machinery and leads to α-synuclein aggregation and the formation of inclusions. In contrast, the S18Y UCH-L1 mutant seems to exert protective effects against α-synuclein aggregation and inclusion formation. Sporadic PD patient midbrain sections have been shown to contain UCH-L1- and α-synuclein double-positive LBs. USP9X and USP8 inhibit the ubiquitination of α-synuclein, while USP9X deubiquitinates mono-ubiquitinated α-synuclein. The role of mono-ubiquitinated α-synuclein is unknown, but it is believed to be more aggregation-prone and potentially results in the formation of α-synuclein-positive LBs. In the case of USP9X, inhibition of the ubiquitination process directs the α-synuclein proteins towards the CMA and its degradation, thus conferring protective effects. OTUB1 has amyloidogenic effects and co-localizes with α-synuclein. Lastly, USP8 deubiquitinates Lys63-linked chains, resulting in decreased clearance and potential aggregation of α-synuclein. Created using www.BioRender.com (accessed on 15 December 2022).

Secondly, in a study by Alexopoulou et al. [58], USP8 was associated with α-synuclein and LB pathology. LB patient brain lysates were observed to have USP8 upregulation in the SN and had substantial co-localization with neurons containing pathological inclusions. USP8 and α-synuclein were shown to co-localize and interact at early endosomes, where α-synuclein ubiquitination and clearance rate was decreased by USP8 overexpression. USP8 deubiquitinated α-synuclein with preference for Lys63-linkages, which is a linkage type that targets proteins for lysosomal degradation. The deubiquitination of α-synuclein increased its half-life, decreased its clearance rate, and increased its toxicity, whereas USP8 KD in a neuronal cell model increased α-synuclein degradation and trafficking mediated by lysosomes (Figure 5). Supporting the association between USP8 and α-synuclein, simultaneous USP8 KD in a α-synuclein-expressing *D. melanogaster* PD model reduced WT and mutant α-synuclein levels as well as rescued the rough eye phenotype and the age-dependent locomotor defects associated with this model [58].

Lastly, it was previously shown that Mfn levels are increased in PINK1 KO and Parkin KO flies and that decreased Mfn levels can rescue the associated phenotypes [36,108,109]. In a study by von Stockum et al. [59], it was found through an RNA interference screen in *D. melanogaster* fly cells that USP8 downregulation correlated with decreased Mfn levels. Here, USP8 overexpression resulted in elongated, accumulated, and clumped mitochondria, whereas USP8 downregulation increased fragmentation. Additionally, USP8 downregulation in PINK1 KO flies rescued the phenotypes of this model including neuronal loss, dopamine levels, climbing ability, life span, and mitochondrial structure and function. Similar results were obtained by pharmacological inhibition of USP8 in the same fly models. The mechanism of action of both USP8 downregulation and inhibition was suggested to be through reduced Mfn levels. Overall, this suggest that USP8 affects PD-associated phenotypes in flies through Mfn levels, independently of Parkin [59].

In conclusion, USP8 has several roles both dependent and independent of Parkin and α-synuclein. It deubiquitinates Lys6-linked ubiquitin chains from Parkin, which accelerates its turnover but is also necessary for efficient translocation to the mitochondria and subsequent mitophagy. However, a Parkin-independent mechanism acting through Mfn has also been suggested. Regarding α-synuclein, USP8 co-localizes with α-synuclein in LBs but also deubiquitinates primarily Lys63-linked chains, leading to decreased α-synuclein clearance and potentially increased α-synuclein aggregation. Although these results suggest that USP8 could be a future drug target, it should be noted that it does not meet the requirements for this due to its role as an essential gene. However, its exclusion is still preliminary.

### 2.6. Ataxin-3 Is a Machado-Joseph Disease-Associated Protein That Inhibits Parkin Self-Ubiquitination by Diverting Ubiquitin onto Itself

The sequence similarity of the catalytic site in the Ataxin-3 protein compared to other DUBs is low, but the overall structure of the catalytic triad is conserved [42]. The catalytic site is found in the N-terminal Josephin domain, whereas the C-terminal of the enzyme contains three ubiquitin-interacting motifs (UIMs) flanking a polyglutamine (polyQ) tract. CAG expansion mutations in this gene leads to elongation of the polyQ tract that is associated with the trinucleotide repeat disease, Machado–Joseph Disease (MJD), also termed spinocerebellar ataxia-3 (SCA3) [62,110]. However, some patients with MJD have been observed to have parkinsonian symptoms, which supports an association between MJD and PD [111]. Additionally, some PD patients have CAG expansions in the Ataxin-3 gene, which might suggest that Ataxin-3 also plays a role in PD [112,113,114].

In a 2011 study by Durcan et al. [61], Ataxin-3 was shown to directly interact with Parkin, which was enhanced by Parkin auto-polyubiquitination irrespective of the catalytic domain of Ataxin-3 and the size of the polyQ tract. Additionally, it was shown that Ataxin-3 decreased the degree of Parkin self-ubiquitination, which was dependent on the active site Cys14 of Ataxin-3 and was exacerbated by polyQ tract expansion. Only the expanded version of Ataxin-3 was shown to promote the clearance of Parkin through autophagy, which was verified in vivo [61]. In a later study by Durcan et al. [60] from 2012, it was shown that rather than deubiquitinating Parkin, Ataxin-3 hydrolyzed the ubiquitin conjugates while they were actively assembled but was unable to hydrolyze already formed chains. Indeed, results suggested that expanded Ataxin-3 regulated the degradation of Parkin by stabilizing the Parkin-E2 enzyme interaction, disrupting the normal association and dissociation cycle, and diverting ubiquitin conjugations away from Parkin and onto itself (Figure 4) [60]. Interestingly, there are discrepancies between studies regarding the effects of Parkin on Ataxin-3 ubiquitination. Tsai et al. [115] found that Parkin not only interacts with the expanded polyQ tract of Ataxin-3 but also leads to its ubiquitination and degradation, which could rescue cells from the aggregation and toxicity associated with the polyQ tract of Ataxin-3 [115]. However, in the study by Durcan et al. [61], Parkin was not observed to affect Ataxin-3 ubiquitination levels. Lastly, a study by Noronha et al. [63], showed that cellular oxidation was increased when the expanded Ataxin-3 mutant or α-synuclein was expressed, which was further exacerbated by their co-expression. However, the expression of normal Ataxin-3 alone or with α-synuclein led to similar oxidation levels as in control or α-synuclein-expressing cells, respectively. Similar tendencies were obtained when the cells were exposed to rotenone or iron, where normal Ataxin-3 decreased the defects associated with α-synuclein expression [63].

Findings from the described studies suggest that Ataxin-3 interacts with Parkin and the E2 enzyme during Parkin ubiquitination, leading to disruption of this process. It is believed that ubiquitin instead is diverted onto Ataxin-3 itself and that this leads to reduced Parkin self-ubiquitination and Parkin activity. This effect was increased when the expanded, MJD-associated Ataxin-3 protein was expressed, resulting in increased Parkin clearance. Additionally, it was found that α-synuclein and expanded Ataxin-3 expression separately increase the oxidation levels of cells, which was further increased by their co-expression. However, a tendency towards protection against α-synuclein expression was observed by WT Ataxin-3. It is evident that Ataxin-3 has diverse roles in cells but plays a different role than expanded Ataxin-3 during physiological and disease conditions. The role of Ataxin-3 in PD is still not fully understood [50], and it is therefore not a suitable drug target before more is known.

### 2.7. USP13 KD Protects Dopaminergic Neurons against α-Synuclein-Induced Neuronal Death

USP13 is a DUB widely distributed in the human body but is mainly found in the cytoplasm and nucleus of cells (UniProt accession number: Q92995). USP13’s association to PD and other neurodegenerative diseases such as Alzheimer’s disease (AD) has been extensively studied by Liu et al. in several papers [64,116,117]. Firstly, the group found that significantly higher levels of USP13 were present in postmortem midbrains of PD patients compared to age-matched, healthy, control subjects, suggesting that the enzyme plays a role in the disease. Subsequent experiments investigated mesencephalic neurons from WT and PARK2 KO mice with USP13 expression or knockdown as well as α-synuclein expression [64]. Here, it was shown that USP13 negatively regulates Parkin ubiquitination, function, and solubility (Figure 5), negatively regulates the level, ubiquitination, and clearance of α-synuclein, and regulates proteasome activity independently of Parkin, which affects dopaminergic neuronal viability and motor performances in animal models. The reduced ubiquitination and clearance of α-synuclein by USP13 was verified in vivo in transgenic mice with expression of mutant α-synuclein. Additionally, it was shown that overexpression of USP13 could antagonize the positive effects of the drug Nilotinib, which is known to increase α-synuclein clearance in this animal model, whereas USP13 KD augmented the effects of the drug [64]. Contrary to these results, Vajhøj et al. [118] did not observe any of the neuroprotective effects of USP13 KD observed by Liu et al. [64]. Instead, in an inducible HA-tagged A53T α-synuclein iPSC model, α-synuclein aggregation was increased without any effects on protein levels upon USP13 KD [118]. In the study by Liu et al. [64], it was also shown that USP13 could regulate proteasome activity independently of Parkin, where USP13 expression, even with KD, could increase proteasome activity in α-synuclein-expressing cells irrespective of Parkin expression. Additionally, USP13 KD protected dopaminergic cells in the SN against neuronal death induced by α-synuclein and also improved motor performance skills in α-synuclein-expressing WT and PARK2 KO mice [64].

Based on the prior results, Liu et al. [116] investigated the effects of several USP13 inhibitors that were chemically based on the non-specific USP10 and USP13 inhibitor, Spautin-1. The produced analogues were more potent USP13 inhibitors, had higher blood-brain barrier (BBB) penetration, and lowered α-synuclein levels with different potencies [116]. Additionally, 26S proteasome activity was shown to be increased in α-synuclein stressed cells, but not in non-stressed cells [117]. One lead candidate was chosen based on potency, and its pharmacokinetics were investigated. The inhibitor was shown to increase α-synuclein ubiquitination, reduce α-synuclein levels, and improve survival of cortex and striatum neurons in A53T α-synuclein mutant mice [116]. In a subsequent study, also by Liu et al. [117], this inhibitor was found to be well-tolerated and safe to use in mice with no visible adverse effects and no sign of tissue toxicity. The inhibitor protected against dopamine loss, rescued motor performances, and increased both α-synuclein ubiquitination and clearance in vivo in α-synuclein-expressing mice but only when USP13 was fully expressed [117].

USP13 has also been shown to regulate protein clearance through other mechanisms and through other E3 ligases than Parkin. Another E3 ligase, NEDD4, can ubiquitinate class III phosphoinositide-3-kinase (PI3K) complexes, which in turn can produce the signaling molecule, phosphatidylinositol-3-phosphate (PI3P). The class III PI3K complex contains VPS15, VPS34, Beclin-1, and Atg14, where VPS34 is ubiquitinated by NEDD4. Ubiquitination of VPS34 leads to its proteasomal degradation and suppression of autophagy. However, NEDD4 has been observed to recruit USP13 upon auto-ubiquitination at its Lys1279, and this complex deubiquitinates VPS34, leading to its stabilization and production of PI3Ps. The produced PI3Ps can be recognized and lead to the recruitment of Atg protein complexes that perform critical steps in autophagosome formation. These steps include conjugation of LC3/Atg8 to phosphatidylethanolamine lipids, expansion of the phagophore by Atg9, and autophagosome closure by Atg2 [91,119].

Overall, recent studies indicate that USP13 has several different roles, including a role in the aggregation of α-synuclein, Parkin function, and proteasome activity, where USP13 KD or inhibition increased α-synuclein ubiquitination and clearance both in vitro and in vivo. Additionally, USP13 KD seemed to enhance the effects of the drug, Nilotinib, whereas USP13 expression mitigated this. USP13 also seemed to positively affect autophagy independently of Parkin. These diverse roles of USP13 encourage further investigation of the DUB and its potential as a therapeutic.

### 2.8. UCH-L1 I93M Mutant Inhibits CMA-Mediated Degradation of α-Synuclein

Ubiquitin carboxyl-terminal hydrolase L1 (UCH-L1), also known as PARK5, is a protein expressed in neurons, testes, and ovaries and is involved in the processing of ubiquitinated proteins by hydrolyzing the peptide bond of the C-terminal glycine of ubiquitin (UniProt accession number: P09936) [69,120]. UCH-L1 is one of the most abundant proteins in the brain, where it accounts for 1–5% of the total soluble protein content [66], and it has a relatively uniform expression pattern that is particularly strong in the dopaminergic neurons of the SN [121]. In line with this, UCH-L1 has been suggested as a possible biomarker for several types of CNS damage and disease, including ischemic brain injury, strokes, epileptic seizure, traumatic and spinal cord injury [78] as well as PD and atypical parkinsonian disorders (APD), in which UCH-L1 levels in the cerebrospinal fluid were decreased [122]. Besides its hydrolase activity, UCH-L1 has been reported to function as a ubiquitin ligase in its dimerized form and appears to ubiquitinate α-synuclein [123]. Additionally, in vivo studies in mice have shown that it stabilizes mono-ubiquitin levels in neurons [124], which was suggested to play a role in synaptic function [125].

UCH-L1 has been shown to be downregulated in AD brains and in idiopathic PD [120], the latter in cases with LB pathology [126]. However, its role in neurodegenerative diseases such as PD and AD is still controversial [69,86,127,128]. Nonetheless, sporadic PD patient midbrain sections have been found to contain α-synuclein- and UCH-L1-double-positive LBs [65,129]. The association between UCH-L1 and α-synuclein is unclear, with some findings suggesting that UCH-L1 overexpression results in α-synuclein accumulation [70] while other studies found UCH-L1 deficiency exacerbated α-synuclein-associated disease [72]. However, one study observed that UCH-L1 suppression in control cells increased α-synuclein aggregation, whereas the same suppression in α-synuclein overexpressing cells resulted in increased clearance, suggesting that UCH-L1 has different roles in physiological and pathological conditions [73].

By sequencing the coding region of the UCH-L1 gene in probands from 72 families with PD, a German family was found with an I93M mutation. This mutation was not in any of the 500 control chromosomes, and the I93 is well-conserved in UCH-L1, the homolog (UCH-L3), and in the rat and mouse orthologues [130]. The mutation was shown to reduce the catalytic activity of the protein [130], increase its interaction with the cytosolic region of LAMP2A (Figure 1c), decrease CMA activity, increase levels of CMA substrates such as α-synuclein [66], increase dopaminergic neurodegeneration in the SN, and decrease dopamine levels in the striatum of mice [131], as well as increase its unfolding and aggregation abilities [70]. Conversely, the S18Y mutation of UCH-L1 has been inversely associated with PD with early disease onset in several populations, suggesting a protective role of this mutation [67,68,132,133,134,135,136,137,138,139,140]. However, meta-analyses and case-control studies in other populations have shown no association between the UCH-L1 protein and PD in general, no association between PD and the I93M mutant, and no protective effects of the S18Y polymorphism [86,128,135,140,141,142,143,144,145,146,147]. Regardless of its association with PD, the I93M mutant was shown to have inclusion formation abilities that were not seen in the WT or S18Y mutants. However, these inclusion formation abilities were decreased with co-expression of the protective S18Y mutant [70]. Similarly, Andersson et al. [69] showed that the unfolding rate of the UCH-L1 protein was highest for the I93M mutant, then the S18Y mutant, and lastly the WT protein. For both mutants, the secondary structure was similar to WT UCH-L1, but the I93M was more unfolded and less stable whereas the S18Y mutant was more compact [69]. The dimerization ability and ligase activity of both mutants were decreased, but the lowest ligase activity was found in the S18Y mutant, which was suggested to confer its protective effects by decreasing α-synuclein ubiquitination and inhibiting the I93M ligase activity in trans. Supporting this, UCH-L1 was shown to preferentially form Lys63-linked ubiquitination, which was suggested to decrease α-synuclein degradation and increase accumulation [123]. However, Andersson et al. [69], suggested that the partial unfolding of the I93M mutant leads to toxicity through the exposure of the hydrophobic surface, which could result increased aggregation and interaction with the CMA machinery (Figure 5). The mutation was also proposed to make the protein more susceptible to chemical modifications associated with PD such as oxidation [69]. Another suggested mechanism for the protective effects of the S18Y mutant was by conferring antioxidant functions in neuronal cells, which was not observed by WT UCH-L1. Here, expression of this mutant decreased the sensitivity of cells towards toxins, independently of its ubiquitin-binding and hydrolase activity abilities [148]. Supporting this, expression of the S18Y mutant exerted protective effects on the dopaminergic neurons in vivo upon toxin treatment [149].

Several post-translational modifications have been observed to significantly affect the function of UCH-L1. Firstly, increased levels of oxidized and carbonyl-modified UCH-L1 were found in brains of AD and PD patients compared to control subjects [120], where reactive metabolites such as endogenous and oxidized dopamine can induce unfolding and aggregation of UCH-L1 and result in decreased cell viability [76,150]. Interestingly, in silico analysis of the interaction between UCH-L1 and a parkinsonism-inducing dopamine derivative was shown to be located around the active site Cys90, and this interaction was displaced away from the active site in the S18Y UCH-L1 mutant, thus suggesting a different protective mechanism of this mutant. Insoluble and catechol-modified UCH-L1 proteins were observed in vivo in the brains of MPTP-treated PD model mice, which further supports the in vitro results indicating a role in the progression of PD [76]. Secondly, the effects of farnesylation of the Cys220 of UCH-L1 have been investigated in a study by Liu et al. [74]. Here, a fraction of UCH-L1 was membrane-bound in both healthy and diseased (PD, AD, and MSA) neurons with co-localization to the ER, which was dependent on farnesylation. Genetic or pharmacological inhibition of the farnesylation decreased the amount of membrane-bound UCH-L1, resulting in reduced α-synuclein accumulation and toxicity both in vivo and in vitro. This suggests that inhibition of UCH-L1 farnesylation could be a potential therapeutic target against α-synuclein-associated PD [74]. However, in a study by Bishop et al. [151], the effects of farnesylation on the subcellular localization of UCH-L1 were dependent on the method of farnesylation inhibition [151]. Cerqueira et al. [75] found that the cytosolic localization of UCH-L1 was implicated in the regulation of Mfn2 levels and affected the mitochondrial function and morphology. Here, UCH-L1 KD reduced Mfn2 levels, altered mitochondrial morphology with enlargement of the organelle, decreased connectivity, lowered branching, disrupted interaction between the mitochondria and ER, lowered mitochondrial calcium uptake, and increased mitochondrial oxygen consumption rate as well as Complex I activity. Overexpression of WT UCH-L1 increased the levels of Mfn2, which were further augmented by expression of a farnesylation-incompetent UCH-L1 mutant that had impaired ability to associate with membranes. These results were verified in a *D. melanogaster* in vivo model [75].

Of therapeutic relevance, Kim et al. [77] found a truncated form of UCH-L1 missing the N-terminal (NT-UCH-L1) that was present in several cell lines and in mouse whole brain and had protective effects in vivo. The truncated form did not display any hydrolase activity, had a more flexible structure, could be mono-ubiquitinated, was degraded by the proteasome, and was more aggregation-prone compared to the full-length UCH-L1. The ubiquitinated form of NT-UCH-L1 was predominantly localized to the cytosol, whereas the non-ubiquitinated version was primarily localized to the mitochondria. Additionally, ubiquitination-competent NT-UCH-L1 had a faster turnover rate compared to full-length UCH-L1, whereas the non-ubiquitinated form had an even faster rate, indicating that mono-ubiquitinations increased the stability of the truncated protein. Of interest, expression of NT-UCH-L1 and of ubiquitination-incompetent NT-UCH-L1 resulted in decreased ROS levels and protected cells against toxin-induced cell death, which was not observed in cells expressing full-length UCH-L1. Lastly, in vivo expression of NT-UCH-L1 was shown to protect nigrostriatal dopaminergic neurons in transgenic mice from the toxicity associated with MPTP treatment. The researchers therefore suggested that NT-UCH-L1 could be used to prevent neurotoxicity in PD through increased levels of this form [77].

In conclusion, the roles of normal UCH-L1 and mutant UCH-L1 are diverse and seem to relate to several aspects of PD. UCH-L1 was suggested to affect α-synuclein accumulation, but its specific role is controversial and believed to be dependent on the conditions. Nonetheless, studies indicate that increased risk of disease is associated with the I93M mutant through various mechanisms, including unfolding, decreased CMA activity, and decreased ligase and hydrolase activity. Conversely, the S18Y mutant was associated with decreased disease risk and neuronal protection through decreased unfolding abilities, antioxidant properties, and decreased ligase activity. The subcellular localization of the protein seems to be important, where its membrane association was associated with increased α-synuclein accumulation and decreased viability while its cytosolic localization was associated with increased Mfn2 levels that could rescue mitochondrial function and morphology defects. The subcellular localization seemed to be regulated by the farnesylation state, and the N-terminal truncated form of UCH-L1 was mainly found in the cytosol and conferred protective effects against ROS and cell death. It is evident that both genetic and non-genetic risk factors affect the function of UCH-L1, making it a particularly difficult therapeutic target against PD. This is further complicated by its wide expression and wide range of functions. However, overexpression of the NT-UCH-L1 truncated version of UCH-L1 could potentially be used to protect against induced oxidative stress.

### 2.9. USP9X Deubiquitinates Mono-Ubiquitinated α-Synuclein

Little is known about the USP9X protein structure apart from its catalytic motif [43], which is highly conserved across species at a level similar to developmental master regulatory genes and has a particular and complex expression pattern during development [127,152]. It has been observed to be essential for embryogenesis and early development, where it is one of the 500 genes with the lowest tolerance to DNA variations [43], and specific deleterious variants are associated with neurological diseases [153]. USP9X interacts with at least 35 proteins, many of which are thought to be targets [43]. The protein is highly expressed in neural stem cells and progenitors of the adult brain, where its expression affects the progenitor organization and fate [154,155]. It has a complex intracellular localization pattern dependent on the cell type and the conditions. The protein is predominantly found in cytoplasmic and membrane-associated puncta [154,156], but other studies have also found it in the mitochondria and nucleus [157,158]. These diverse localizations are consistent with its diverse functions, which are regulated by its availability in different compartments [43]. Lower USP9X levels have been observed in the cytosolic fraction of PD patient SN lysates compared to controls [79].

Regarding PD, USP9X has been shown to co-localize with α-synuclein-containing LBs in PD patient brains [79]. LB-associated α-synuclein is usually mono-ubiquitinated, but the physiological role of this ubiquitination is unknown. The α-synuclein mono-ubiquitination is performed by the E3 ligase, SIAH, which is also present in the LBs, whereas USP9X mediates its deubiquitination. In vitro studies have shown that mono-ubiquitinated α-synuclein is degraded by the proteasome, while its deubiquitination by USP9X results in degradation by the less effective autophagy pathway [79]. Decreased degradation makes the protein more prone to aggregation, oligomerization, and formation of α-synuclein fibrils, which is associated with PD [159]. In an MPTP-induced PD mouse model, USP9X expression was upregulated in all parts of the brain, pointing towards a cellular response to the toxicity [160]. However, studies have shown that PD patients experience reduced levels of USP9X, which is suggested to result in the accumulation of the more aggregation-prone mono-ubiquitinated α-synuclein (Figure 5). Indeed, Rott et al. [79] showed that purified USP9X reduced the level of α-synuclein mono-ubiquitination, which reduced the number of α-synuclein inclusions and decreased toxicity, whereas USP9X KD increased this. It was suggested that substances with the ability to activate USP9X or inhibit α-synuclein mono-ubiquitination could facilitate autophagic degradation of α-synuclein and function as therapeutics in cases of α-synuclein-associated PD [79].

Collectively, the role of USP9X in PD and α-synuclein clearance is somewhat controversial as it is suggested to have both positive and negative effects on α-synuclein clearance. USP9X is known to deubiquitinate mono-ubiquitinated α-synuclein, which is both suggested to lead to accumulation due to degradation by the less effective autophagy pathway and to increase the clearance of α-synuclein. Therefore, the effect of α-synuclein mono-ubiquitination and deubiquitination should be more fully elucidated. Its utilization as a therapeutic target could be a possibility, but the diverse results on its function as well as its roles during embryogenesis should be considered.

### 2.10. USP14 Is a Negative Regulator of the Proteasome, Autophagy, and Mitophagy

The USP14 protein is known to be associated with the 26S proteasome, which is necessary for its activation [80]. USP14 inhibits the degradation of several proteins by the proteasome through its deubiquitinating ability towards Lys48-linked ubiquitin chains on proteasome-associated substrates [81,82]. However, the DUB becomes catalytically active upon binding with the 26S proteasome [80], and this interaction, together with the binding of ubiquitin chains on the substrate, facilitates gate-opening of the 20S proteasome and increased proteolysis [83]. The opposing regulation of the proteasome by USP14 is believed to enhance selectivity [83]. However, the expression of USP14 leads to proteasomal inhibition and accumulation of specific proteins independently of its catalytic activity [81,161].

**Figure 6:**
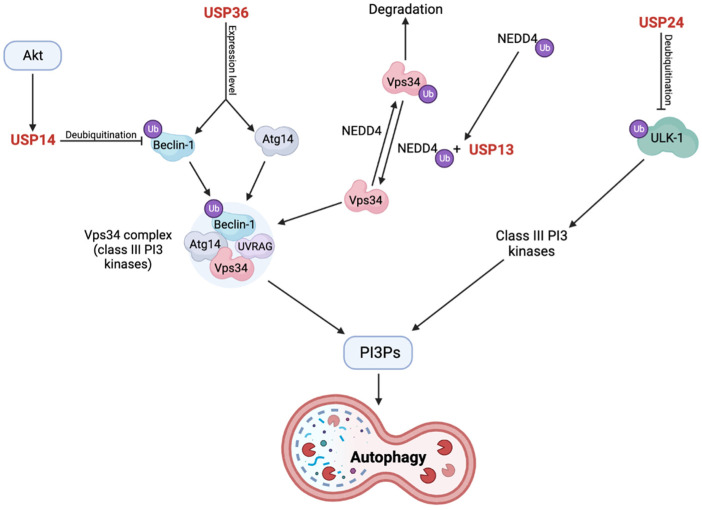
Visualization of how USP13, USP14, USP24, and USP36 affect the regulation of autophagy through PI3Ps. USP14 is activated by Akt-mediated phosphorylation, which results in USP14-mediated deubiquitination of Beclin-1. Beclin-1 in its ubiquitinated form interacts with Atg14 and UVRAG in the Vps34 complex, which is a class III PI3 kinase that produces PI3P from PI. PI3Ps in turn aid in autophagy by mediating autophagosome formation. USP14 hereby acts as a negative regulator of autophagy. USP36 increases the expression level of Beclin-1 and Atg14, leading to increased autophagy through the formation of the Vps34 complex and formation of PI3Ps. USP13 forms a complex with NEDD4, which can deubiquitinate Vps34 and increase the formation of the Vps34 complex, inducing autophagy through the formation of PI3Ps. Lastly, USP24 inhibits autophagy by deubiquitinating ULK-1, which positively regulates other class III PI3 kinases and aids in the autophagy process. USP24 is hereby a negative regulator of autophagy, although its association to PD is still somewhat controversial. Created using www.BioRender.com (accessed on 15 December 2022).

USP14 has been shown to negatively regulate autophagy, where USP14 KD or inhibition resulted in increased number of autophagy vesicles, autophagosomes, and autolysosomes, increased mitochondrial fragmentation, reduced mitochondrial volume, and increased association between the 20S proteasome and USP14 [84]. The mechanism of action of USP14 in autophagy was discovered in a study by Xu et al. [82]. Here, USP14 negatively regulated autophagy in a Beclin-1- and Akt-dependent manner. USP14 was activated by phosphorylation by Akt, which resulted in the Lys63-linked deubiquitination of Beclin-1. Normally, ubiquitinated Beclin-1 increases autophagy through increased interaction with Atg14 and the UV radiation resistance-associated gene (UVRAG) protein, which are important regulators of the VPS34 complex. This interaction between Beclin-1 and VPS34 was increased in cells without USP14 compared to those with USP14 [82]. The VPS34 complex is a class III PI3K that converts PI into PI3P, as described earlier, and PI3P is an activator of autophagy by affecting autophagosome formation (Figure 6) [82,162]. The association between Akt and USP14 was further verified by the treatment of cells with insulin-like growth factor 1 (IGF1) or epidermal growth factor (EGF), which are known activators of Akt. Treatment with IGF1 or EGF reduced autophagy, and this was restored upon inhibition of Akt and could not be observed in USP14 KD cells [82]. Nonetheless, the role of USP14 in autophagy is still somewhat controversial. In a study by Kim et al. [163], USP14 was shown to play a dual role in both UPS and autophagy, where the two pathways act oppositely with USP14 as the common ground. Here, when USP14 was inhibited, the proteasome became active and the autophagic flux decreased, thereby making USP14 a positive regulator of autophagy [163].

USP14 was also observed to affect mitophagy in a study by Chakraborty et al. [84], where USP14 KD or inhibition resulted in increased mitophagy, which was dependent on pro-fission protein, Drp1, and fusion protein, Mfn2. This was verified by diminished mitochondrial clearance after USP14 inhibition upon simultaneous KO of Drp1 and Mfn2. Interestingly, this effect on mitophagy was shown to be independent of PINK1 and Parkin KO in vitro (Figure 4). Furthermore, USP14 KD or inhibition could rescue or improve climbing ability, mitochondrial respiration, dopamine levels, mitochondrial morphology, and life span of *D. melanogaster* flies with either PINK1 or Parkin KO [84].

When investigating the therapeutic potential of USP14, the small molecule 1-[1-(4-fluorophenyl)-2,5-dimethylpyrrol-3-yl]-2-pyrrolidin-1-ylethanone (IU1), which selectively binds to the active form of USP14 on the proteasome, has been found [81,84]. This inhibitor had no toxicity in *D. melanogaster* flies or neuronal populations, but its ability to penetrate the BBB is yet to be determined [84]. IU1 has similar abilities to induce autophagy as UPS activity, with EC_50_ values of 63.4 µM and 62 µM, respectively [82], and it might mediate mitophagy in a Prohibitin2-dependent manner [84]. Additionally, IU1 was shown to decrease the phosphorylation and ubiquitination level of tyrosine hydroxylase (TH), where the former was suggested to result in TH degradation [164]. Banerjee et al. [165] evaluated IU1 in a rat PD model and found that USP14 levels changed with age, with increased levels in the SN and cerebellum. This is important for the investigations of the distributive abilities and dosage of IU1 and could indicate that some brain regions are more susceptible to IU1 than others. This study also showed that the choice of toxins used to induce PD in the rodent model is important as some toxins increased USP14 expression in the SN, whereas other did not, which is relevant for the usage of IU1 as a drug [165].

In summary, USP14 regulates the activity of the proteasome by deubiquitinating targeted substrates but also by increasing the proteolytic activity of the proteasome upon association. This is believed to enhance specificity, but it still inhibits proteasomal degradation overall. USP14 also negatively regulates both autophagy and mitophagy, which are dependent on Beclin-1 deubiquitination as well as Drp1 and Mfn2, respectively. More is yet to be discovered about USP14 and its diverse roles in other neurological diseases such as AD [165,166], but investigation of the therapeutic value and inhibitors of USP14 is already ongoing and potentially holds great promise for the future.

### 2.11. USP24 Negatively Regulates Autophagy through Destabilization of ULK-1

USP24 is located at the so-called PARK10 locus, which contains several genes and is linked to late-onset PD in the Icelandic population [87]. The gene promoter of USP24 contains a Nuclear Factor kappa-light-chain of activated B cells (NFkB) binding site, where increased NFkB levels or activity upon stress or inflammation was shown to increase USP24 promoter activity and gene expression [167].

Although USP24 was recently found to be a negative regulator of autophagy [85], its association with PD is still unclear with opposing results from a variety of genetic and associative studies in different populations [86,87,88,89,90]. Under the assumption that single-nucleotide polymorphisms (SNPs) in USP24 do affect predisposition and/or the age of onset of PD [86,88], its mechanism in doing so was still unknown and poorly characterized until recently [85]. Lipinski et al. [168,169] have conducted several studies on autophagy regulation and found that USP24, amongst others, regulates autophagy. Here, USP24 KD led to autophagosome accumulation and increased levels of LC3-II [169]. Similar results were obtained in a more recent study [85], where USP24 was a negative regulator of autophagy by regulating the formation of autophagosomes upstream from the lysosomes. Interestingly, USP24 was shown to function in a converging pathway to USP14 with PI3Ps as a common denominator. As described, PI3Ps are produced by PI3Ks, which in turn can be positively regulated by the ULK-1 protein kinase complex. USP24 negatively regulated the expression level and activity of ULK-1 through deubiquitination, where USP24 KD was shown to increase the stability and decrease the degradation of ULK-1 without affecting the complex at the transcriptional level. Collectively, this indicates that ULK-1 is needed for USP24-dependent regulation of autophagy, which downstream affects the activity of PI3Ks. These results were confirmed in iPSC-derived dopaminergic neurons. The authors also found associations between USP24 expression levels and USP24 SNPs in PD patients compared to controls [85].

The present data indicate that USP24 is a negative regulator of autophagy through deubiquitination and inhibition of ULK-1, which in turn activates class III PI3Ks, produces PI3Ps, and subsequently mediates autophagy. However, the association between USP24 and PD is still controversial based on several association studies, which necessitates further investigation of this DUB.

### 2.12. Loss of USP36 Causes Impairments in the Mitophagy Process through Altered Expression Levels of Autophagy Complex Proteins

Deubiquitinating enzyme USP36 is non-specifically expressed and widely distributed in humans (UniProt accession number: Q9P275).

Geisler et al. [91] performed a high-content imaging screen of a siRNA DUB library on Parkin translocation to depolarized mitochondria. Here, USP36 was found to be important for Parkin translocation to depolarized mitochondria, which was dependent on its catalytic activity. This was further verified by the fact that USP36 KD impaired CCCP-induced mitophagy and ubiquitination of mitochondrial proteins, which could be rescued by its reintroduction. These effects were suggested to be direct results of the diminished Parkin translocation rather than deubiquitination of Parkin targets (Figure 4). The mechanism behind this could be increased activity of the long form of phosphatase and tensin homologue (PTEN), a phosphatase of Ser65 phosphorylated ubiquitin (pS65-Ub), which is a perquisite for effective translocation and activation of Parkin. Indeed, USP36 KD resulted in increased long form PTEN protein levels as well as reduced pS65-Ub levels, suggesting that USP36 affects the expression of this protein [91]. Geisler et al. [91] also showed that mRNA expression levels of several genes were significantly affected by USP36 KD, where 528 genes were upregulated, and 491 genes were downregulated. Of the autophagy complex proteins, Beclin-1 and Atg14 were the only genes with reduced expression levels. Based on these study results, it was suggested that the presence of a phosphorylation-competent Atg14 was sufficient to rescue the activity of VPS34-Beclin-1-Atg14 complex in cells depleted of USP36. Supporting this, expression of the S29A Atg14 mutant was associated with reduced mitophagy after prolonged treatment with CCCP, which could be rescued by both WT and phosphorylation mimicking (S29D) Atg14. Overall, Geisler et al. suggested that USP36 does not affect cytosolic processes during mitophagy but rather disturbs the process at the transcriptional level, specifically by altering Atg14 and Beclin-1 levels (Figure 6) [91].

Collectively, USP36 seems to be important in the effective translocation of Parkin, in the mitophagy process, and in the expression of necessary autophagy complex proteins. The effects of USP36 loss were first suggested to be due to increased expression of the phosphatase, PTEN, upon USP36 KD. However, USP36 seems to affect the expression level of autophagy complex proteins Beclin-1 and Atg14 as these were reduced upon USP36 KD, and this may have led to the impairments in the mitophagy process.

### 2.13. OTUB1 Has Amyloidogenic Properties and Co-Localizes with α-Synuclein in LBs

Otubain-1 (OTUB1) belongs to the deubiquitinating enzyme family called ovarian tumor (OTU) domain cysteine protease superfamily and is capable of specifically removing Lys48-linked ubiquitin chains from its substrate [40,170].

In silico analysis by Kumari et al. [92] identified differential expression of 52 proteins in LBs of PD compared to control cells, including OTUB1, which was shown to have amyloidogenic properties. In this study, OTUB1 amyloid aggregates could be found after exposure to heat-induced or oxidative stress-induced conditions where particle size increased over time from monomers to oligomers and finally to fibrils. The potential spreading of these three forms was investigated in SH-SY5Y cells; the monomers were found evenly distributed in the cells, but the oligomers and fibrils were internalized and found in punctate structures. Interestingly, the oligomer-containing punctates were observed to heavily disrupt the actin filament of the cytoskeleton. Treatment with the oligomers was more toxic than treatment with the two other forms, where the oligomers led to increased ROS production and showed signs of pore-like structures in the cytoplasmic region. Treatment with oligomers or fibrils reduced the mitochondrial membrane potential and increased cell surface roughness, whereas the monomers did not show the same effects. As mentioned, during stress conditions, it was shown that OTUB1 co-localized with both α-synuclein and the pathological pS129 α-synuclein, which were increased in expression together with OTUB1 during rotenone treatment [92]. Similarly, in a study by Xia et al. [171], OTUB1 was enriched with α-synuclein in LBs. Kumari et al. [92], showed that treatment of cells with both the oligomer and fibril form of OTUB1 enhanced the expression of both forms of α-synuclein. The same co-localization between OTUB1 and pS129 α-synuclein was observed in the hippocampus and cortex of a rotenone-induced PD mouse model, which also exhibited increased expression of both proteins in the same areas [92].

The fact that oxidative stress conditions could induce OTUB1 aggregation was in line with observations showing that excess amount of ROS and reactive nitrogen species can modify proteins and lead to their aggregation [172]. Interestingly, a later study by Kumari et al. [93] confirmed that the rotenone-induced oxidative stress conditions resulted in S-nitrosylation at Cys23 and Cys91 of OTUB1, leading to reduced catalytic activity, defects in the protein structure, compromised binding with the E2 enzyme, Ubc3, and increased aggregation tendencies. These S-nitrosylations were confirmed both in vitro in SH-SY5Y cells and in rotenone-treated mouse brains [93].

These data suggest overall that OTUB1 has amyloidogenic and cytotoxic properties through S-nitrosylation of Cys23 and Cys91, resulting in disruption of the cytoskeleton and increased ROS production in vitro. These effects can potentially induce the disease mechanism of PD and could be either the cause or the result of co-localization with α-synuclein in LBs. The different types of OTUB1 have different degrees of cytotoxicity, suggesting that the production of a molecule that can inhibit the formation of OTUB1 oligomers and fibrils could have therapeutic effects in PD cases where OTUB1 expression is altered.

### 2.14. The Role of USP40 in PD Is Still Controversial

The role of USP40 in PD is still largely controversial as some studies found an association between SNPs in the gene and PD patient populations, while others did not [86,88,90]. USP40 is associated with several other diseases such as cancer [173,174] and inflammatory bowel disease [175], but its mechanism of action in PD has not yet been established.

## 3. Discussion, Conclusion, and Future Perspectives

Oxidative stress has been implicated in neurodegenerative diseases including PD, in which the accumulation of free radicals and ROS could contribute to dopaminergic neuronal loss in the SNpc of PD patients [176]. An important process in the limitation of oxidative stress is the maintenance of mitochondrial health through mitophagy, which is a complex process with several layers of regulation in which DUBs play a significant role. Other pathological processes are also implicated in PD, such as protein aggregation, defective proteasome regulation, impaired autophagy, and alterations in protein expression. DUBs also play a significant role here and can be both protective or disruptive to the cells and result in the PD phenotype. In this paper, a variety of DUBs have been described in terms of their function in PD and their potential roles as drug targets. It is evident that from all the available DUBs in the human genome, only a few seem to be implicated in the mitophagy pathway and in the pathogenesis of PD.

Of the described DUBs, USP15, USP30, and USP35 impaired mitophagy by deubiquitinating mitochondrial proteins targeted by Parkin, whereas USP33 and USP8 deubiquitinated Parkin itself, which affected Parkin activity and translocation to the mitochondria. On the contrary, USP8 function was observed to be necessary for efficient translocation and function of Parkin although it also resulted in increased Parkin turnover. However, a negative role of USP8 was also suggested as it was shown to deubiquitinate α-synuclein, resulting in increased α-synuclein levels and decreased clearance. Additionally, decreased USP8 expression was associated with decreased Mfn levels, whereas USP8 overexpression had negative effects on mitochondrial morphology. Similarly, USP9X deubiquitinated α-synuclein; however, the exact effects on α-synuclein clearance and aggregation are still unclear. As with USP33 and USP8, the MJD-associated Ataxin-3 reduced the ubiquitination of Parkin by diverting the actively assembling ubiquitin molecules onto itself instead of onto Parkin, which was suggested to reduce activity and turnover of Parkin, but negatively affecting its function. The MJD-associated polyQ tract expansion was observed to enhance the deubiquitinating ability of Ataxin-3 and further mitigate Parkin function. Both USP14 and USP36 affect the autophagy protein complex and hereby regulate autophagy and mitophagy. USP14 is a negative regulator of both and inhibits mitophagy in a PINK1/Parkin-independent manner as well as regulates the proteasome and increases proteolysis. On the other hand, USP36 is a positive regulator of mitophagy and autophagy through promotion of Parkin translocation and regulation of autophagy protein expression levels, respectively. In a somewhat similar manner as USP14, USP24 also negatively regulates and inhibits autophagy. USP13 has been shown to have several functions, where it negatively regulates Parkin function, α-synuclein aggregation, and proteasome activity, whereas USP13 KD or inhibition seemed to improve some of these defects. Lastly, UCH-L1 is a protein that interacts with the CMA machinery and co-localizes with α-synuclein in LBs. The CMA interaction was increased by the I93M mutation, resulting in decreased CMA activity and decreased α-synuclein turnover. The effect of this mutation is suggested to be due to increased unfolding, potential aggregation of the mutant, and/or α-synuclein aggregation. Conversely, the S18Y mutant has been suggested to play a protective role as it is inversely associated with PD. The subcellular localization of UCH-L1 was affected by farnesylation, which affected α-synuclein accumulation, cell viability, Mfn2 levels, and mitochondrial morphology and function. Similar to the I93M UCH-L1 mutant, OTUB1 has amyloidogenic properties through S-nitrosylations, which disrupt the cytoskeleton, increase ROS production, and lead to cytotoxicity. These effects were greatest for OTUB1 in its oligomeric form, less in the fibrillary state, and non-toxic as a monomer. Finally, USP40 plays a controversial role in PD with differing results from a variety of association studies. However, an interesting case-control study [86] investigating the synergistic effects of USP24, USP40, and UCH-L1 polymorphisms on PD risk in a Taiwanese population showed that a specific genotype of USP24 alone reduced PD susceptibility in subjects over the age of 60 years. When considering the whole population without age stratification, however, USP24 acted synergistically protective with specific genotypes of both USP40 and UCH-L1 [86]. This indicates that while the association of several DUBs to PD is still unclear, they might play synergistic roles with other genes or environmental factors, perhaps explaining the discrepancies between the different association studies.

It is evident that the DUBs associated with PD have diverse roles in the disease, and several even produce disease defects independently of their deubiquitinating abilities. These differing abilities could allow drug targets that affect the disease-causing events without disrupting the deubiquitinating ability that might play important roles in other functions. Examples of this could be inhibition of oligomerization of OTUB1 or specific inhibition of CMA interaction by the I93M mutant UCH-L1. However, in cases where the deubiquitinating ability of DUBs is crucial to the disease-causing or disease-protecting event, their inhibition or augmentation should be carefully considered. Generally, the deubiquitinating events performed by these DUBs might be important and carefully balanced in a variety of pathways or have different effects in other tissues or in other settings, which can be both healthy and pathological. Therefore, not all DUBs might be applicable drug targets. However, several of the DUBs already have inhibitors under investigation and are believed to be potential drug targets against PD. These DUBs include USP30, USP14, and USP13. It is important that the full role of the DUBs in the whole body is elucidated before therapeutic-directed inhibitors or activators can be developed. Additionally, several other therapeutic aspects for a disease such as PD should be considered, including administration method, distribution of the drug in the body (i.e., can the drug pass the BBB and enter the brain), the metabolism and elimination of the drug, and the potential side effects. Although these are important considerations, PD is a progressive and devastating disease with no cure and less than satisfactory symptom management. This allows for compromises in the administration method, side effects, and other limitations as symptoms and disease progression must be weighed against these.

Much is still unknown about PD, and further research on its pathomechanism is of great interest. PD is undoubtedly a complex disease, and the investigation into the DUBs points towards an even more complex interplay between disease-causing genes, genetic risk factors, and the environment. Which one of these specific roles the DUBs play is still unknown. However, this additional layer of regulation of many of the well-known pathways implicated in PD opens new therapeutic avenues for the future. This could allow the development of targeted therapy against the implicated gene, combination therapy with other drugs for an improved and synergistic effect, or fine-tuning of only slightly dysfunctional pathways. The understanding of the DUBs is the next step towards decoding and hopefully improving treatment of the disease.

## Figures and Tables

**Table 1:** Table of the DUBs described in this review, their chromosomal localization, and their functional relation to PD.

Protein Name	Chromosomal Location	PD-Related Function
**USP15**	12q14.1	Inhibits Parkin-dependent mitophagy by deubiquitinating Parkin targets [45].
**USP30**	12q24.11	Inhibits Parkin-dependent mitophagy by deubiquitinating Parkin targets [46,47,48,49]. Controversial whether it affects Parkin recruitment [46,47,50]. USP30 inhibitors are under investigation [51,52,53,54,55,56].
**USP33 (VDU1)**	1p31.1	Inhibits mitophagy by interacting with and deubiquitinating Parkin at Lys435, which decreases its stability and translocation [57].
**USP35**	11q14.1	Suggested to associate with and stabilize Mfn2, which inhibits Parkin-mediated mitophagy [47].
**USP8**	15q11.2-q21	Increases Parkin degradation by deubiquitinating Parkin, but also increases mitophagy as deubiquitination is necessary for efficient Parkin activation and translocation [50]. Deubiquitinates α-synuclein, which reduces its clearance [58]. Overexpression has negative effects on mitochondrial morphology, but KD rescues this through decreased Mfn levels [59].
**Ataxin-3**	14q32.1	Reduces Parkin self-ubiquitination by diverting ubiquitin onto itself, leading to increased Parkin degradation [60]. This function is increased by MJD-associated polyQ tract expansions [61,62]. Mutant increased oxidation levels, exacerbated by α-synuclein expression [63].
**USP13**	3q26.33	Reduces Parkin and α-synuclein ubiquitination [64]. Regulates proteasome activity independently of Parkin [64]. KD protects TH+ neurons against α-synuclein-induced neuronal death in vivo [64].
**UCH-L1**	4p14	UCH-L1 and α-synuclein-positive LBs in sporadic PD patient midbrain sections [65]. I93M mutant is associated with autosomal dominant PD [66,67,68,69,70,71]. I93M mutant has increased unfolding and stronger interaction with CMA machinery, which inhibits CMA and leads to α-synuclein accumulation [66,67,68,69,70,71]. However, still complex role in α-synuclein clearance and accumulation [70,72,73]. Potential protective effects of S18Y mutant [66,67,68,69,70,71]. Membrane association increased by farnesylation, which increases α-synuclein accumulation [74]. Cytosolic localization is associated with increased Mfn2 levels [75]. Reduced hydrolase activity, increased aggregation, and reduced cell viability when conjugated with oxidized dopamine [76]. N-terminal deficient version might be protective against cellular oxidation [77]. Potential biomarker for PD and other CNS-related diseases [78].
**USP9X**	Xp11.4	Reduced levels are observed in PD patients [79]. Deubiquitinates the more aggregation-prone mono-ubiquitinated α-synuclein, which directs towards autophagy [79].
**USP14**	18p11.32	Regulates proteasome activity by deubiquitinating proteasome-bound substrates [80,81,82,83]. Binding to the proteasome and activation through ubiquitin binding results in proteasomal gate-opening and proteolysis [80,81,82,83]. Affects autophagy by regulating Beclin-1 ubiquitination [82]. Inhibits mitophagy in a Drp1- and Mfn2-dependent, but PINK1/Parkin-independent manner [84].
**USP24**	1p31.1	Negative regulator of autophagy in a p53- and mTOR-independent manner, but dependent on ULK-1 and class III PI3Ks [85]. Association to PD is controversial [86,87,88,89,90].
**USP36**	17q25.3	Positively affects Parkin translocation upon mitochondrial depolarization [91]. Affects ubiquitination of TOM70 and Mfn1, but not by disrupting Parkin-mediated ubiquitination [91]. Decreased expression levels of autophagy complex proteins, Beclin-1 and Atg14, upon KD [91].
**OTUB1**	11q13.1	Has amyloidogenic properties and forms amyloid aggregates under pathological conditions [92]. The oligomeric aggregates disrupt cytoskeleton, induce cytotoxicity, increase ROS production, show signs of pore-like structures in the membrane, and co-localize with both phosphorylated and unphosphorylated α-synuclein [92]. Aggregation is believed to be due to S-nitrosylation [93].
**USP40**	2q37.1	Controversial association with PD. Mechanism of action is unknown [86,88,90].
